# Nanocomposite Metamaterials Based on Self-assembled Titanium Dioxide Rolls with Embedded Gold Nanoparticles

**DOI:** 10.1038/s41598-019-43588-7

**Published:** 2019-05-07

**Authors:** Stella Kutrovskaya, Alexey Kucherik, Anton Osipov, Vlad Samyshkin, Alexander Istratov, Alexey V. Kavokin

**Affiliations:** 1 School of Science, Westlake University, 18 Shilongshan Road, Hangzhou, 310024 Zhejiang Province China; 2 Institute of Natural Sciences, Westlake Institute for Advanced Study, 18 Shilongshan Road, Hangzhou, 310024 Zhejiang Province China; 3 Department of Physics and Applied Mathematics, Stoletov Vladimir State University, 600000 Gor’kii street, Vladimir, Russia

**Keywords:** Synthesis and processing, Electronic devices, Computational science

## Abstract

An experimental method for fabrication of a nanocomposite metamaterial based on a self-assembly of titanium dioxide microtubes with encapsulated gold nanoparticles (NPs) is proposed. The formation of microtubes is induced by laser irradiation in the presence of an external magnetic field. It is shown that the variation of the metal NP concentration leads to the increase of the optical absorption of the metamaterial. The possibility of using arrays of oriented microtubes as absorbing n-doped layers for solar cells is demonstrated.

## Introduction

The development and tailoring of spatially ordered metamaterials is one of the main goals of the modern nanotechnology. The self-assembly is one of the most promising methods of fabrication of such metamaterials. This method has already demonstrated its high potentiality for fabrication of various micro- and nanostructures^1^, superlattices^2^, supercrystals^3^ etc.

The photonics is one of the most important application areas for self-assembled nanostructures. The optical response of metamaterials composed by periodically modulated nanostructures is governed by the geometry of these structures rather than by refractive indices of their components. The nanophotonics is aimed, in particular, to the development of perfect absorbers that would absorb 100% of the incident light in a selected narrow spectral range^4,5^. It also targets the fabrication of wide-band absorbers for photovoltaic applications^6,7^. An array composed by multilayer microtubes represents a metamaterial that may find its application in wide-band hyperlenses^8^. Clearly, the development of growth and fabrication methods for realization of spatially ordered nanostructures with tailored geometrical parameters is an important goal of the modern nanophysics.

Nowadays, nanocrystals of titanium dioxide (TiO_2_) are being intensively studied in many laboratories worldwide. The interest to this nanomaterial is caused by its unique physical properties and functionalities^9,10^. TiO_2_ films are photosensitive, they possess both chemical and mechanical stability and exhibit unusual optical properties. Depending on their oxygen fraction the oxides of titanium (Ti_x_O_y_) demonstrate the properties of metals or semiconductors and may possess high refractive indices. This makes them promising for multiple optical applications. In addition, TiO_2_ nanoparticles synthesized by a laser ablation method are characterized by high concentrations of surface defects that affect the energies of donor and acceptor bound states located in the vicinity of the conduction and valence bands, respectively. Due to the presence of these impurities, the free carrier concentration may vary in large limits in TiO_2_ nanoparticles^11^.

It should be noted that structures based on titanium oxide can effectively absorb light only in the narrow UV range because of their huge energy band gaps. To enhance the absorption in the visible spectral range, TiO_2_ films are usually doped with non-metallic anions or transition metal cations^12^. The implantation of metal nanoparticles (NPs) with prominent plasmon resonances into a semiconductor thin film matrix leads to a change of the dielectric permeability at optical frequencies, the variation of conductivity and other optical and electronic effects^13^. The metamaterials composed by TiO_2_ and metal NPs offer an opportunity of wide tuning of their optical and electronic properties by variation of the concentration of the encapsulated metallic NPs^14^.

The present study is aimed at the formation of multilayer TiO_2_ microtubes by self-organization from thin films synthesized by laser ablation in air, in the presence of spatially inhomogeneous magnetic fields. We experimentally observe the layered structure of the self-organized microtubes. We show that the absorption spectra of the synthesized microtubes are strongly sensitive to the concentration of metal NPs in the oxide matrix. The synthesized nanocomposite metamaterial is promising for photovoltaic applications as an effective n-layer with a tailored spectral dependence of the absorption coefficient.

## Results

### The method of Formation of multilayer TiO_2_ microtubes

**Figure 1:**
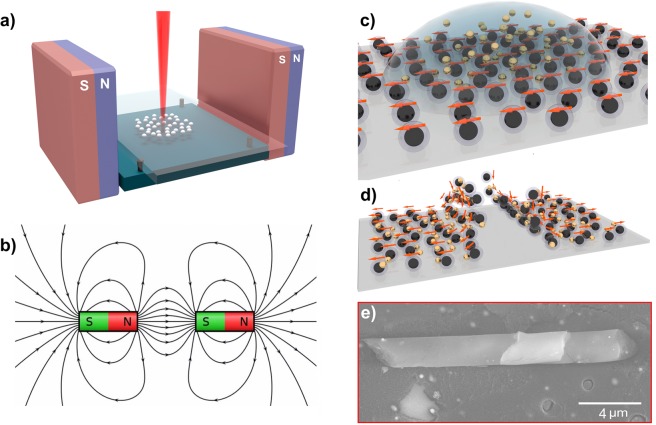
The experimental setup for the deposition of thin films of titanium oxide composed by Ti-TiO_2_ nanoparticles with use of the laser ablation technique (**a**,**b**) the schematic distribution of the magnetic field lines; (**c**,**d**) is the schematic presentation of the procedure of introduction of gold NPs and the self-induced formation of microtubes by rolling thin films of titanium dioxide. The formation of microtubes is controlled by the spatially homogeneous magnetic field. (**c**) The schematic orientation of the magnetic momenta of Ti-TiO_2_ NPs on the surface of the substrate in the presence of the external magnetic field with (**d**) showing the subsequent disorientation of magnetic momenta due to the effect of the gradient of the magnetic field on the magnetically oriented NPs. This disorientation induces the breaks in the thin film which triggers the rolling process leading to the formation of multilayer microtubes. (**e**) The schematic images are drawn not in scale; (**e**) shows the SEM image of the microtube.

We have used the laser ablation method for the synthesis of metastable forms of Ti_x_O_y_. Thin films of titanium oxide have been deposited on a solid transparent substrate by means of the fast cooling of a melted phase of Ti_x_O_y_ created by laser ablation of a titanium target in air^15,16^. A 30 µm laser beam has been used to scan the surface of a bulk titanium target. over the beam has been moved along the circles having a diameter of 3 mm (see Fig. 1a). The scanning has been realized with steps of 30 µm. Each spot has been illuminated during 10 seconds in order to deposit a layer of the thickness of 200 nm or larger.

As a result of its interaction with the laser irradiation characterized by the wavelength of 1.06 µm, the pulse duration of 100 ns, the pulse repetition rate of 20 kHz and the energy per pulse of 1 µJ the target has been partially melted and a vapor-gas stream directed opposite to the light beam has been generated. The propagation of titanium plasma in air triggers formation of oxide shells covering the ablated titanium nanoparticles. The collection of the resulting material on the surface of the substrate has been achieved by a direct vapor deposition in the presence of a spatially homogeneous magnetic field created by two rectangular (the length of 40 mm, the height −of 30 mm) NdFeB magnets characterized by the magnetic induction of 500 mT and placed at the distance of 10 mm from each other. The distance between magnets was chosen so that the magnetic field lines could be approximated by unclosed curves with quasi infinite radii in the area of the laser action (see Fig. 1b). The magnetic field effect on the tube formation exhibited a threshold behavior. We observe that the magnetization induced by an external field of 100–400 mT in a titanium oxide layer of 200 nm thickness is too weak for triggering the stable formation of the microtubes. The field of 500 mT efficiently breaks the film and the microtubes are formed, as Fig. 1d shows. The further increase of the field affects trajectories of the ablated Ti particles and makes their flying distances much longer. As a consequence, the deposition takes place far from the homogeneous magnetic field area. In this regime, the array of self-assembled microtubes loses its regularity and becomes strongly inhomogeneous. We have also optimized the distance between the titanium target and the collector to ensure the sufficient oxidation of ablated particles in the air. The oxidation is essential to secure the homogeneity of the deposited film. The optimized target-collector distance is of the order of 2 mm in the employed set-up^17^.

The self-assembly of microtubes is triggered by the external magnetic field (see the schematic in Fig. 1с,d). Titanium is an anomalous paramagnetic material whose magnetic susceptibility increases with the temperature increase^17,18^. The deposited NPs possess metallic nuclei that form macroscopic magnetic domains oriented by the external magnetic field^19^, as Fig. 1с shows. Once the magnetic field is switched off, the disorientation of magnetic moments of titanium nuclei leads to the formation of ferromagnetic domains. Domain walls are subject to a mechanical strain that competes with a weak adhesion of the thin film of titanium oxide to the surface.

Once a critical strain value is achieved, this film breaks, and the attraction of magnetic NPs leads to its rolling, as Fig. 1d schematically shows. Finally, multilayer microtubes of titanium oxide are formed, as the scanning electron microscopy (SEM) images certify (see Fig. 1e). After deposition on a solid substrate the film was immediately wetted by a colloidal solution of gold NPs with an average diameter of 10 nm. We have used the CW-laser ablation method for creation of a colloidal solution containing gold NPs^20^. In our experiments, the NP concentration was ranging from 0.05 up to 1 μg/ml. It varied during the fabrication process due to the variation of a mass ratio between the solvent and the dispersed phase. We have taken advantage of the drop-off technique developed for the deposition of metallic NPs^21^. NPs penetrate into a porous crystal matrix of titanium oxide. The application of external magnetic field of 500 mT resulted in breaking of the deposited film along the domain walls. The subsequent rolling of the tubes resulted in the formation of a regular array of titanium oxide microtubes with embedded gold NPs.

### The microscopy and optical studies of self-assembled microtubes

**Figure 2:**
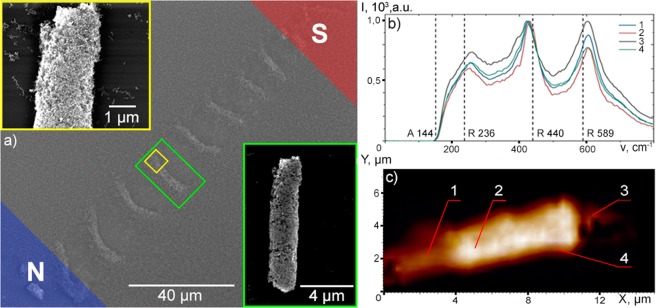
The results of spectral and structural characterisation of TiO_2_ microtubes. (**a**) Shows the SEM image of a 1D array of the microtubes oriented normally to the magnetic field lines. The poles of the stationary magnet are marked as N and S, for the northern and southern poles, respectively. The images are taken at different magnifications (Mag) in order to reveal the porous structure of a microtube: the green box corresponds to a SEM image of the full tube at Mag 10000х and the yellow box shows the SEM image of the end of the same tube at Mag 40000х; (**b**) shows the normalised micro Raman images taken at different spots of the microtube indicated by (1, 2, 3, 4) on the AFM image. (**c**) The number and the location of Raman intensity peaks *I*(*v*) are signatures of the molecular structure of the object.

We have studied the structure and composition of self-assembled TiO_2_ microtubes oriented by the spatially inhomogeneous magnetic field (see Fig. 2a) with use of the atomic force microscope (AFM) Ntegra Spectra combined with a Raman spectroscopy set-up. The set of the Raman spectra of the studied metasurface is shown in Fig. 2b. For the excitation we have used a 5 mW CW-laser light with a wavelength λ = 473 nm. The laser beam has been focused by use of the long-focus objective 100 × 0.7 NA.

This analysis shows that self-assembled microtubes consist of the textured porous titanium dioxide. Remarkably, we do not observe the characteristic main anatase peak^22^ at the frequency of 144 cm^−1^ (indicated by the dashed A-line in Fig. 2b), while the features of the other polycrystalline phase of titanium oxide, rutile, are present at 236, 440 and 589 cm^−1^, marked as the R-lines in Fig. 2b. These features are shifted with respect to the central frequencies of the most intensive rutile lines at 260, 427 and 605 cm^−1^, respectively^23^. The coexistence of the red and blue shifts of different Raman lines can be ascribed to the deviations from the stoichiometry and to the presence of a specific kind of nonstoichiometric defects, that appear due to the laser irradiation of a titanium target. The defects cause broadening of the Raman modes that is very apparent starting from the shoulder below 200 cm^−1^. These data appear to be in a good agreement with the literature. Namely, according to ref.^24^, the titanium oxide films of the width of less than 500 nm are entirely amorphous, while rutile is the preferential phase of TiO_2_ nanoparticles of the diameter over 50 nm^25^.

**Figure 3:**
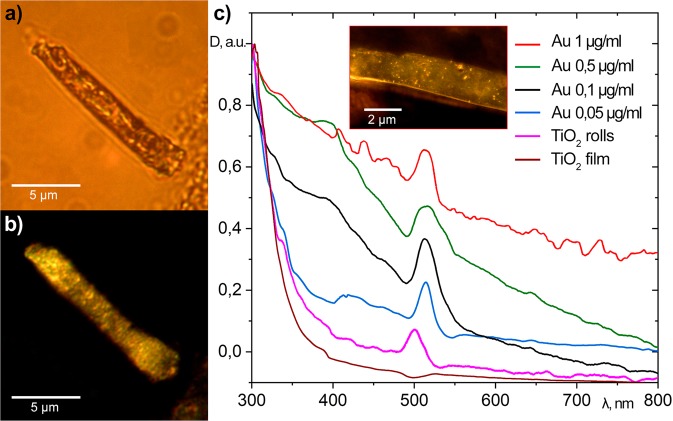
Illustrates the optical properties of the self-assembled microtubes with encapsulated gold NPs. (**а**) An optical image of the tube obtained by using the optical microscopy with (**b**) showing the corresponding dark-field image; (**с**) shows the experimental absorption spectra of the array of titanium dioxide microtubes deposited on a glass substrate. The spectra are taken at different concentrations of the gold NPs, ranging from 0 (pink curve) to 1 μg/ml (red curve). D (λ) is the optical density. The brown curve shows the reference absorption spectrum of a homogeneous TiO_2_ film deposited on the same substrate. The high resolution dark field image of a part of the microtube covered with gold NPs is shown in the inset. The image (**b**) is excited by a light beam focused in the bulk of the microtube, while the image in the inset to (**c**) is obtained by focussing the excitation light on the surface of the microtube.

The multilayer structure of studied microtubes is revealed in the images obtained with use of the dark-field microscope with a 100х objective (Fig. 3a,b). Note, that the total area of the sample corresponding to this image is 50 × 50 µm. The intensity of light scattered by the structure is spatially inhomogeneous. Namely, inside the microtubes we observe a non-uniform distribution of the dark-field signal that appears as a result of the density fluctuations (see Fig. 3b and the inset to Fig. 3c). It is important to note that the parts of the film that are not rolled to microtubes give zero contributions to the dark-field microscope signal.

Gold nanoparticles that were embedded into the wide-bandgap semiconductor microtubes demonstrate the Mie scattering in a wide range of wavelengths: from the red to the green colour. If the system is irradiated by light, the conduction band electrons in gold NPs are being photoexcited to higher energy states. This makes them capable for overcoming the Schottky barrier and entering the titanium oxide crystal matrix^26^. Clearly, the gold NPs enhance the photoelectric activity of the titanium dioxide in several ways. Namely, they boost the absorption coefficient of the system due to the Purcell and nanoantenna effects, they help charge separation in the semiconductor by the direct electron transfer as well as by the plasmon-induced charge transfer, they help reducing the radiative losses by suppressing the radiative recombination rate in a semiconductor due to the induction of stationary electromagnetic fields^27^.

**Figure 4:**
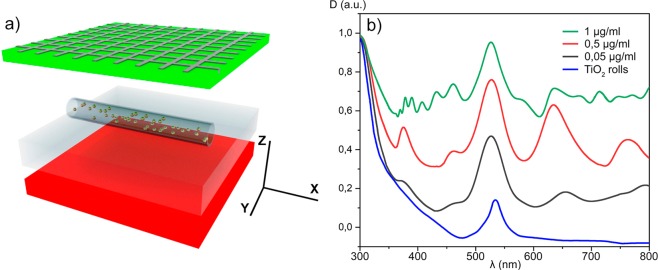
The spectral modelling of the optical properties of TiO_2_ microtubes. (**a**) Schematics of the computational domain that consisted of a glass substrate with TiO_2_ microtubes and gold NPs. A plane wave source is situated below the glass substrate. It is shown as a red box. The detectors are located in the grid nodes above the air region (shown as a green box). The grid step is assumed to be 10 nm. (**b**) The calculated optical density D (λ) shown in (**a**) depends on the gold NP concentration that varies from 0.05 up to 1 μg/ml. The blue curve shows the calculation result for the case of a titanium oxide microtube containing no gold NPs.

The measured absorption spectra D (λ) show the characteristic features of titanium dioxide microtubes with embedded gold NPs (Fig. 3c). For comparison, a reference absorption spectrum of a planar film is shown. One can see that rolling of the films to the array of microtubes significantly affects its absorption spectrum. In particular, a supplementary strong absorption peak forms at the wavelength of about 500 nm. We attribute this peak to a plasmon resonance. It is characteristic of the rolled structure based on the 200 nm-wide film^28^. The peak position slightly varies with the increase of the concentration of gold NPs. We also observe that the increase of the concentration of gold NPs encapsulated into the titanium oxide matrix leads to the increase of the absorption coefficient in the entire visible spectral range (VSR): from 400 up to 780 nm. In addition, once the concentration of gold NPs achieves 1 µg/ml the absorption spectra start exhibiting local minima. This reflects the energy transfer between the localised surface plasmon resonance in a metal and the optically induced electron-hole plasma in a semiconductor. The non-radiative energy transfer is known in metal-semiconductor systems^26,29,30^. Theoretically, it can be described with use of the finite difference time domain method (FDTD)^31^. Adapting this method to our system we describe the optical properties of metals within the Drude-Lorentz model^32^. We consider a model system composed of gold NPs, embedded in a microtube of titanium dioxide (see Fig. 4a). The distance between metal NPs along X, Y, Z axes is varied between 0 and 100 nm. The nodes are assumed to be filled with gold spheres randomly. The top and the bottom of the simulation volume are limited by the perfectly matched layers (PML) with the thicknesses of 100 nm, whereas the periodic boundary conditions are set at the side walls. The laser source is assumed to be located below the substrate. The laser polarization is assumed to be directed along the OX axis, so that the light source provides a plane wave front parallel to the XOY-plane. The calculations are performed for 10 optical cycles. The transmission is defined as the ratio of the intensity of light in the plane coinciding with the bottom of the upper PML to the incident light intensity. In all cases, the calculated spectra are quite similar (see Fig. 4b). The variations obtained for different locations of gold spheres do not exceed 5%.

The calculation results are presented in Fig. 4b. The calculated spectra clearly demonstrate that the admixture of gold NPs strongly affects absorption properties of the films. The coupling between neighbouring NPs increases with the increase of the NP concentration, which is why the characteristic localized plasmon resonance in the range of 520–540 nm^33^ becomes more and more pronounced, in full agreement with the experimental data shown in Fig. 3c. The shifts of the width and the position of the experimentally observed peaks are not reproduced by the model. They can be attributed to the spatial inhomogeneity of microtubes that is not accounted for in our calculation. The model assumes an ideal periodicity of the layered structure that is not fulfilled in the experiment. The ideal periodic structure is characterised by absorption peaks at about 370, 460, 640 and 760 nm that are not observed experimentally.

**Figure 5:**
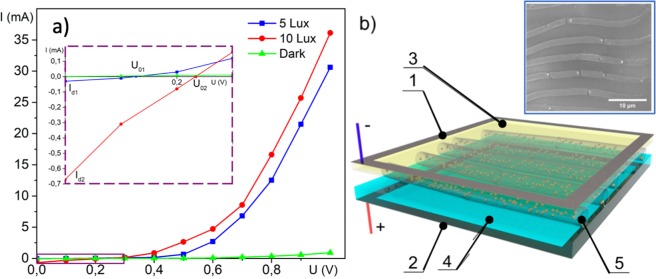
The photocurrent measurements in a solar cell based on TiO_2_ microtubes. (**а**) Shows the experimental current-voltage characteristics of the tested solar setup taken at different illumination power: the blue curve corresponds to the illumination power of 5 Lux and the red curve corresponds to the illumination power of 10 Lux. The reference green curve is taken in dark (0 Lux). The inset presents the enlarged violet box located at the left bottom angle of the plot; (**b**) shows schematically the structure of the studied solar element: 1, 2 are the top and bottom contacts, 3 is the upper transparent electrode, 4 is a silicon layer (p-type), 5 represents the titanium oxide microtubes doped with gold NPs (n-type). Their orientation and size can be estimated from the inset showing the SEM image of the structure.

Both the data and the model analysis show the high potentiality of the realised composite metamaterials for the use in solar cells (see Fig. 5). For testing the transformation of the incident irradiation to the electric current we have employed the setup shown schematically in Fig. 5b. The microtubes of titanium dioxide have been formed on a surface of the glass substrate holding metallic contacts at its edges. This top element plays a role of the n-type semiconductor. As the p-type bottom element we have used a 500-nm thick polycrystalline silicon film fabricated by the magnetron sputtering method. The choice of polycrystalline silicon has been made in order to enlarge the functional spectral range of the resulting solar cell. Effectively, the titanium oxide absorbs light in the UV and visible range, while the radiation of the wavelengths of 800 nm and longer passes through the upper element and excites holes in the silicon film. We expect this combination to be favourable for the improvement of the quantum efficiency of our photovoltaic device.

The current-voltage curve characterizing our p-n junction is characterized by the dark current the corresponds to the drift of minority carriers in the vicinity of the junction. A sufficient concentration of electrons is injected into the oxide in the presence of the irradiation by light. As one can conclude from Fig. 5, the tested structure is characterised by the intrinsic photo-electromotive force and a short-circuit current. This explains the existence of zero-voltage current and zero current at U = 0.16 V and the light power of 5 Lux (also at U = 0.23 V and the light power of 10 Lux). Under the effect of the irradiation, the diffusion carrier current (I_d_) is induced due to the generation of additional carriers in the p-n junction region. The current is caused by holes, mostly. The variation of the light intensity leads to the variation of the photocurrent. The current-voltage curves taken at different light intensities demonstrate a similar non-linear behaviour. We anticipate that higher values of the photocurrent are achievable if using a semiconductor with a lattice constant closely matching one of the bottom element of the cell. Overall, these measurements show the high potentiality of TiO_2_ microtubes for photovoltaic applications^34^.

## Conclusions

Optical metamaterials based on the titanium dioxide find multiple applications in various areas of photonics. The enhancement of the plasmonic resonance has been observed in multilayer structures of TiO_2_ with embedded gold NPs. The system is highly promising for the realisation of a new generation of solar cells. Our work presents the experimental method for realisation of multilayer microtubes based on the titanium oxide. The developed laser ablation process allows controlling the film thickness that provides us with an efficient tool for variation and control of the optical properties of synthesised metamaterials. This is achieved due to the strong dependence of the optical absorption spectra of these microtubes on the concentration of gold NPs. We show that the gold NPs boost the absorption coefficient of the system, in general. Clearly, the inclusion of metal NPs characterised by plasmon frequencies that depend on the sizes of the NPs enables one to strongly improve the quantum efficiency of a photovoltaic device based on titanium oxide microtubes.
